# Increasing rate of hospitalization for severe peptic ulcer in digestive disease emergencies after the pandemic

**DOI:** 10.1097/MD.0000000000031716

**Published:** 2022-12-02

**Authors:** Chenxing Jian, Zili Zhou, Chunkang Yang, Ning Zhao, Haijun Bao, Shengbo Han, Jinhuang Chen, Xiaogang Shu

**Affiliations:** a Department of Minimally Invasive Surgery, Affiliated Hospital of Putian University, Teaching Hospital of Fujian Medical University, Putian, Fujian, China; b Department of Gastrointestinal Surgery, Union Hospital, Tongji Medical College, Huazhong University of Science and Technology, Wuhan, Hubei, China; c Department of Gastrointestinal Surgery, Sichuan Provincial People’s Hospital, University of Electronic Science and Technology of China, Chengdu, Sichuan, China; d Department of Gastrointestinal Surgical Oncology, Fujian Cancer Hospital and Fujian Medical University Cancer Hospital, Fuzhou, Fujian, China; e Department of Emergency Surgery, Union Hospital, Tongji Medical College, Huazhong University of Science and Technology, Wuhan, Hubei, China.

**Keywords:** emergency, medical strategy, pandemic, peptic ulcer

## Abstract

Since December 2019, the novel coronavirus has spread worldwide, affecting more than 510 million people, with more than 6 million deaths. However, some of the potential effects of the pandemic have not been thoroughly studied. We collected data from 2 regional emergency centers from May to November for the years 2015 to 2019, before the pandemic, and from May to November 2020, after the pandemic. We evaluated the incidence of each major type of digestive disease before and after the pandemic in adults at the 2 hospitals, which experienced coronavirus disease 2019 outbreaks with varying severity. A total of 11,394 patients were enrolled in the study Affiliated Hospital of Putian University (PUTIAN, n = 5503) Union Hospital, Tongji Medical college, Huazhong University of Science and Technology (UNION, n = 5891), and the proportion of male patients was approximately the same at both hospitals, with 3360 (61.1%) and 3680 (62.5%), respectively. The average ages of the patients were 55.8 ± 18.4 years PUTIAN and 54.3 ± 15.8 years UNION. The numbers of patients at the 2 hospitals increased steadily, but in 2020, the number of patients at UNION declined. The baseline characteristics of the 2 groups at the 2 hospitals showed significant differences for age before and after the pandemic but not for sex. The constituent ratios of diseases in each year in the 2 hospitals differed. The number of patients with peptic ulcers in 2020 was significantly different from those in each year from 2015 to 2019 (PUTIAN 2015‐2020, 15.0%, 18.2%, 14.9%, 16.9%, 19.5%, 34.9%; UNION 2015‐2020, 29.2%, 32.5%, 29.3%, 29.4%, 29.7%, 41.3%, respectively). The rates of peptic ulcer increased dramatically in both hospitals in 2020. An increase in the incidence of severe peptic ulcer was observed after the pandemic compared to the same period before the pandemic. Therefore, these factors should be considered in the formulation of public health strategies and the allocation of medical resources in the post pandemic era.

## 1. Introduction

Since December 2019, the novel coronavirus has spread worldwide,^[1,2]^ affecting more than 510 million people, with more than 6 million deaths. Severe acute respiratory syndrome coronavirus 2 (SARS-CoV-2) infection can affect multiple organs and result in mortality^[3–13]^; it can also indirectly affect other aspects of health systems.^[14]^ Scholars believe that pandemics can affect mental health, economic status, job opportunities and other social factors.^[15]^ A study^[16]^ from Germany showed that the pandemic also changed the emergency medical situation, as the numbers of cholecystectomies and appendectomies in the pandemic year were significantly lower than those in the reference year of 2019. However, it is unclear whether such effects will be sustained in the post pandemic era.

In the post pandemic era, people will continue to experience great psychological pressure, and such pressure may induce the development of many diseases, which should attract the attention of medical personnel. After April 8, 2020, the medical activities in medical institutions in Wuhan, which was the most severely affected area in China, gradually returned to normal. However, during the recovery period, some changes were observed, such as the number of visits and the proportions of visits for certain diseases. Such data are important for the allocation of medical resources after a pandemic. The present study primarily focused on changes in the characteristics of emergencies, particularly those involving the digestive system, after the pandemic was controlled. We also compared the situations in 2 different regions. At present, few articles have reported similar experiences in the field of emergency surgery. We intend to share our experiences with the hope that they will help our colleagues around the world in the post pandemic period.

## 2. Methods

### 2.1. Study design

This was a retrospective study to determine whether severe peptic ulcers increased after the pandemic. Since there was a certain upward trend in the total number of visits, to avoid bias, we selected the proportion of peptic ulcers in patients with digestive system diseases as the object of comparison between different years.

We hypothesized an increase in severe peptic ulcer patients after the pandemic. During the pandemic, from December 2019 to April 2020, severe social restrictions were imposed in Wuhan. People were isolated in their homes, and only a very small number of people could continue to work, such as medical staff, volunteers and people with other jobs that could not be shut down. Hospitals had few non- coronavirus disease 2019 patients. Therefore, to avoid the bias caused by time period, we only included data from May to November of each year for annual comparison. The study included patients with digestive system diseases admitted to the emergency system and statistically analyzed whether there was a significant difference in peptic ulcer patients in different years.

### 2.2. Inclusion criteria

We collected data on the following digestive system diseases from both hospitals: acute appendicitis, acute pancreatitis, biliary tract inflammation, bowel obstruction or bleeding, esophageal and gastric variceal bleeding, hernia, liver abscess or cancer, peptic ulcer, and trauma. Due to different pathogenic factors, esophageal and gastric variceal bleeding and peptic ulcer bleeding were analyzed separately. Peptic ulcer was defined as a severe peptic ulcer with bleeding, obstruction, and perforation. Gastric cancer was analyzed separately. Biliary tract inflammation included tumors, stones and inflammation of the biliary tract due to other causes. Bowel obstruction or bleeding included postoperative intestinal obstruction below the jejunum, primary intestinal obstruction, and intestinal bleeding. Trauma was defined as injury to a digestive system organ caused by various mechanisms. Liver abscess or cancer was defined as a liver abscess, liver rupture due to cancer, or liver cancer with severe abdominal pain. Hernia was defined as a hernia in the abdominal area involving the digestive system.

### 2.3. Exclusion criteria

We excluded patients with digestive diverticula, achalasia, Dieulafoy disease, intra-abdominal tuberculosis, and gastroliths from our study.

### 2.4. Data collection

We collected data from the 2 hospitals Affiliated Hospital of Putian University (PUTIAN); Tongji Medical College, Huazhong University of Science and Technology (UNION), Union Hospital, Tongji Medical College, Huazhong University of Science and Technology) during the same period from May to November for the years 2015 to 2019.

### 2.5. Statistical analysis

SPSS 20.0 software was used to analyze the data. The chi-square test was used for count data, and analysis of variance was used for measurement data. Bonferroni correction was applied to analyze the peptic ulcer composition ratio in 2020 compared with those in each year from 2015 to 2019.

### 2.6. Ethical approval

All methods in this study were performed in accordance with the Declaration of Helsinki. The data were anonymous, and the requirement for informed consent was therefore waived. The protocol of this study and informed consent exemption were approved by the Ethics Committee of Putian University Affiliated Hospital (No. 2021028).

## 3. Results

**Figure 1.**
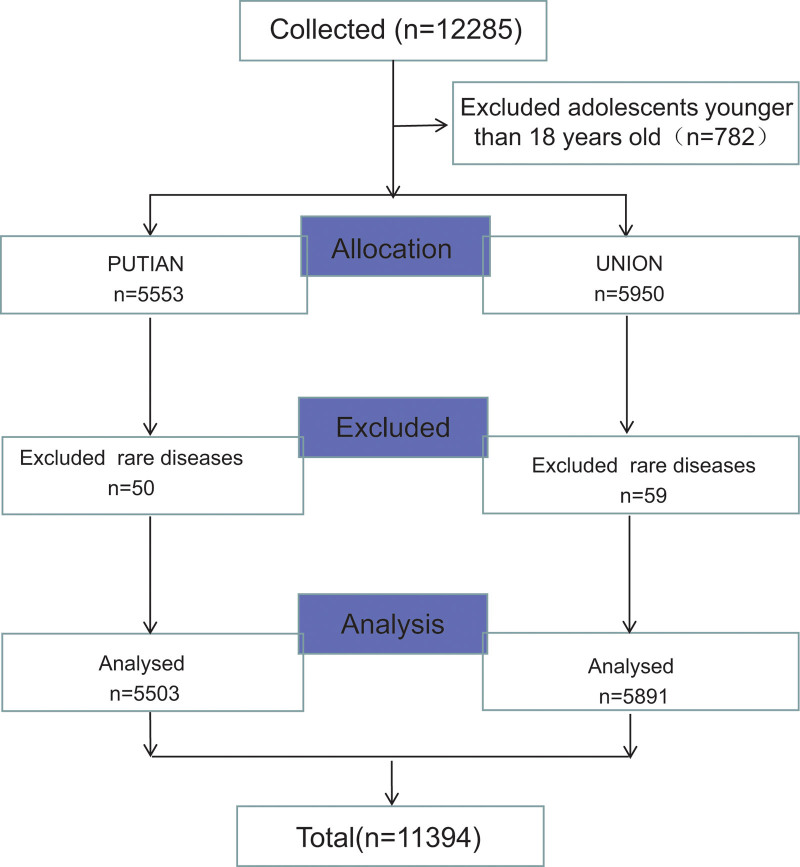
Research flow diagram.

Finally, we obtained the data of 11,394 cases in 12,285 patients (shown in Fig 1). A total of 11,394 patients were enrolled in the study (PUTIAN, n = 5503) (UNION, n = 5891), and the proportion of male patients was approximately the same at both hospitals, with 3360 (61.1%) and 3680 (62.5%), respectively. The average ages of the patients were 55.8 ± 18.4 years PUTIAN and 54.3 ± 15.8 years UNION. From 2015 to 2019, the total number of emergency digestive system-related cases in the 2 hospitals increased steadily, but in 2020, the number of cases in UNION declined; however, the same was not observed in PUTIAN, which did not experience a lockdown during the same period. This may be explained by the fact that medical practice at UNION did not completely return to normal after the lockdown (shown in Table 1). In the 2 hospitals, from 2015 to 2020, there were no significant differences in the sex ratio for each year, but there were significant differences in the age compositions, as shown in Table 1. There seemed to be a trend of increasing age among patients. The baseline characteristics of the 2 groups at the 2 hospitals showed significant differences for age before and after the pandemic (*P* = .002, *P* = .031) but not for sex (*P* = .088, *P* = .944), as shown in Table 2.

**Table 1:** The sex and age distribution of the 2 hospitals in different years (n, %).

	2015	2016	2017	2018	2019	2020	*P*
PUTIAN	795 (14.4)	840 (15.3)	818 (14.9)	930 (16.9)	972 (17.7)	1148 (20.9)	.104
Sex							
Male	455 (57.2)	513 (61.1)	486 (59.4)	582 (62.6)	598 (61.5)	726 (63.2)	.000
Female	340 (42.8)	327 (38.9)	332 (40.6)	348 (37.4)	374 (38.5)	422 (36.8)	
Age (yr)	54.1 ± 18.5	54.4 ± 18.4	53.0 ± 18.8	56.8 ± 18.2	58.0 ± 18.0	57.3 ± 18.3	
UNION	708 (12.0)	827 (14.0)	1009 (17.1)	1107 (18.8)	1318 (22.4)	922 (15.7)	
Sex							.258
Male	446 (63.0)	537 (64.9)	646 (64.0)	683 (61.7)	793 (60.2)	575 (62.4)	
Female	262 (37.0)	290 (35.1)	363 (36.0)	424 (38.3)	525 (39.8)	347 (37.6)	
Age (yr)	53.4 ± 16.8	52.0 ± 15.6	54.1 ± 16.0	54.9 ± 15.8	55.4 ± 15.2	55.4 ± 15.7	.000

PUTIAN = Affiliated Hospital of Putian University, UNION = Union Hospital, Tongji Medical College, Huazhong University of Science and Technology.

**Table 2:** Demographic characteristics.*

	2015‐2019	2020	*P*
PUTIAN			
Sex			.088
Male	2634 (60.5)	726 (63.2)	
Female	1721 (39.5)	422 (36.8)	
Age (yr)	55.4 ± 18.4	57.3 ± 18.3	.002
UNION			
Sex			.944
Male	3105 (62.5)	575 (62.4)	
Female	1864 (37.5)	347 (37.6)	
Age (yr)	54.1 ± 15.8	55.4 ± 15.7	.031

PUTIAN = Affiliated Hospital of Putian University, UNION = Union Hospital, Tongji Medical College, Huazhong University of Science and Technology.

**Figure 2.**
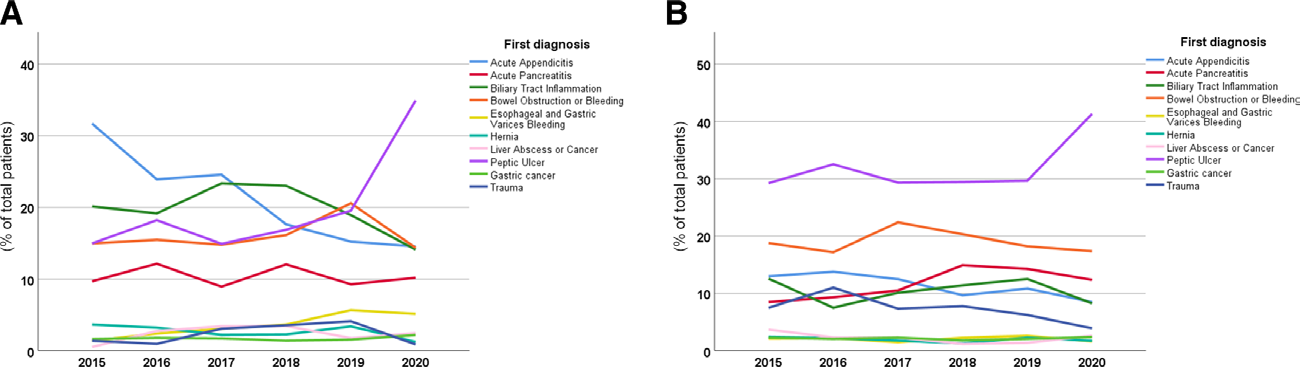
The changing trend of the disease composition ratio in different years in PUTIAN (A) and UNION (B). PUTIAN = Affiliated Hospital of Putian University, UNION = Union Hospital, Tongji Medical College, Huazhong University of Science and Technology.

As shown in Tables 3 and 4, statistical analysis revealed that the constituent ratios of the diseases of interest in each year in the 2 hospitals were different. As seen in Table 3 and Figure 2A, from 2015 to 2020, the numbers of cases of other diseases of interest, except peptic ulcer, generally showed mild fluctuations, but the constituent ratio did not change significantly. Interestingly, in 2020, there was a sudden and significant increase in the proportion of peptic ulcer cases (2015‐2020, 15.0%, 18.2%, 14.9%, 16.9%, 19.5%, 34.9%, respectively) in PUTIAN. The same phenomenon also occurred in UNION, where in 2020, the proportion of peptic ulcer cases also suddenly and sharply increased (2015‐2020, 29.2%, 32.5%, 29.3%, 29.4%, 29.7%, 41.3%, respectively), while it had remained stable or fluctuated slightly in previous years (shown in Table 4 and Fig. 2B).

**Table 3:** The disease composition ratio in different years※ [PUTIAN (n, %)].

	2015 (n = 795)	2016 (n = 840)	2017 (n = 818)	2018 (n = 930)	2019 (n = 972)	2020 (n = 1148)
Acute appendicitis (n = 1133)	252 (31.7)	201 (23.9)	201 (24.6)	164 (17.6)	148 (15.2)	167 (14.5)
Acute pancreatitis (n = 571)	77 (9.7)	102 (12.1)	73 (8.9)	112 (12.0)	90 (9.3)	117 (10.2)
Biliary tract inflammation (n = 1072)	160 (20.1)	161 (19.2)	191 (23.3)	214 (23.0)	184 (18.9)	162 (14.1)
Bowel obstruction or bleeding (n = 885)	119 (15.0)	130 (15.5)	121 (14.8)	150 (16.1)	200 (20.6)	165 (14.4)
Esophageal and gastric varies bleeding (n = 204)	11 (1.4)	20 (2.4)	25 (3.1)	34 (3.7)	55 (5.7)	59 (5.1)
Hernia (n = 142)	29 (3.6)	27 (3.2)	18 (2.2)	21 (2.3)	33 (3.4)	14 (1.2)
Liver abscess or cancer (n = 132)	4 (0.5)	23 (2.7)	28 (3.4)	32 (3.4)	17 (1.7)	28 (2.4)
Peptic ulcer (n = 1142)	119 (15.0)	153 (18.2)	122 (14.9)	157 (16.9)	190 (19.5)	401 (34.9)
Gastric cancer (n = 95)	13 (1.6)	15 (1.8)	14 (1.7)	13 (1.4)	15 (1.5)	25 (2.2)
Trauma (n = 127)	11 (1.4)	8 (1.0)	25 (3.1)	33 (3.5)	40 (4.1)	10 (0.9)

PUTIAN = Affiliated Hospital of Putian University.

※ *P* < .001, There were significant differences in the composition ratio of disease types in each year.

**Table 4:** The disease composition ratio in different year§ [UNION (n,%)].

	2015 (n = 708)	2016 (n = 827)	2017 (n = 1009)	2018 (n = 1107)	2019 (n = 1318)	2020 (n = 922)
Acute appendicitis (n = 660)	92 (13.0)	114 (13.8)	126 (12.5)	107 (9.7)	143 (10.8)	78 (8.5)
Acute pancreatitis (n = 710)	60 (8.5)	77 (9.3)	106 (10.5)	165 (14.9)	188 (14.3)	114 (12.4)
Biliary tract inflammation (n = 620)	89 (12.6)	62 (7.5)	102 (10.1)	126 (11.4)	165 (12.5)	76 (8.2)
Bowel obstruction or bleeding (n = 1126)	133 (18.8)	142 (17.2)	226 (22.4)	225 (20.3)	240 (18.2)	160 (17.4)
Esophageal and gastric varices bleeding (n = 122)	15 (2.1)	18 (2.2)	15 (1.5)	25 (2.3)	34 (2.6)	15 (1.6)
Hernia (n = 113)	17 (2.4)	18 (2.2)	18 (1.8)	14 (1.3)	30 (2.3)	16 (1.7)
Liver abscess or cancer (n = 123)	26 (3.7)	19 (2.3)	23 (2.3)	13 (1.2)	18 (1.4)	24 (2.6)
Peptic ulcer (n = 1870)	207 (29.2)	269 (32.5)	296 (29.3)	326 (29.4)	391 (29.7)	381 (41.3)
Gastric cancer (n = 125)	16 (2.3)	17 (2.1)	23 (2.3)	20 (1.8)	27 (2.0)	22 (2.4)
Trauma (n = 422)	53 (7.5)	91 (11.0)	74 (7.3)	86 (7.8)	82 (6.2)	36 (3.9)

UNION = Union Hospital, Tongji Medical College, Huazhong University of Science and Technology.

§ *P* < .001. There were significant differences in the composition ratio of disease types in each year.

**Figure 3.**
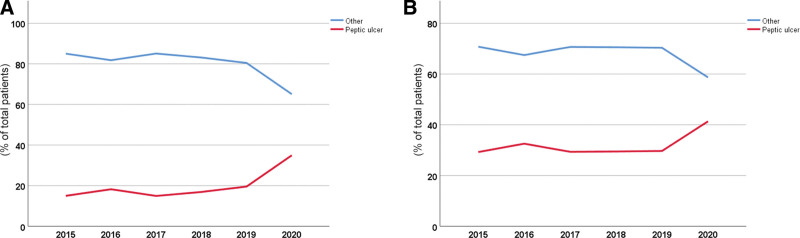
Comparison of peptic ulcer and other diseases in different years in PUTIAN (A) and UNION (B). PUTIAN = Affiliated Hospital of Putian University, UNION = Union Hospital, Tongji Medical College, Huazhong University of Science and Technology.

In view of the above results, we conducted further statistical analysis and compared the peptic ulcer incidence with the total incidence of all other diseases before and after the pandemic, as shown in Table 5, Table 6, Figure 3A, and Figure 3B. The peptic ulcer proportion in 2020 was significantly different from those in each year from 2015 to 2019. Therefore, considering the statistical results together with the number of patients, we believe that the peptic ulcer incidence increased dramatically in both hospitals in 2020.

**Table 5:** Comparison of peptic ulcer and other diseases in different years (n, %).#

	2015	2016	2017	2018	2019	2020	*P*
PUTIAN							
Peptic ulcer	119 (15.0)	153 (18.2)	122 (14.9)	157 (16.9)	190 (19.5)	401 (34.9)	<.001
Others	676 (85.0)	687 (81.8)	696 (85.1)	773 (83.1)	782 (80.5)	747 (65.1)	
UNION							
Peptic ulcer	207 (29.2)	269 (32.5)	296 (29.3)	326 (29.4)	391 (29.7)	381 (41.3)	<.001
Others	501 (70.8)	558 (67.5)	713 (70.7)	781 (70.6)	927 (70.3)	541 (58.7)	

PUTIAN = Affiliated Hospital of Putian University, UNION = Union Hospital, Tongji Medical College, Huazhong University of Science and Technology.

# The Bonferroni method was used for multiple comparative analyses, and there was a significant difference in the composition ratio between 2020 and each year from 2015 to 2019.

**Table 6:** Comparison of peptic ulcers and other diseases in different groups (n, %).

	2015–2019	2020	*P*
PUTIAN			<.001
Peptic ulcer (n = 1142)	741 (17.0)	401 (34.9)	
Others (n = 4361)	3614 (83.0)	747 (65.1)	
UNION			<.001
Peptic ulcer (n = 1870)	1489 (30.0)	381 (41.3)	
Others (n = 4021)	3480 (70.0)	541 (58.7)	

PUTIAN = Affiliated Hospital of Putian University, UNION = Union Hospital, Tongji Medical College, Huazhong University of Science and Technology.

## 4. Discussion

Regarding the demographic data in this study, the ratio of males to females was similar in both hospitals. As seen in Table 2, the average age of patients in the 2 hospitals was similar, with a seemingly increasing trend. Geographically, the 2 hospitals are important medical centers in their respective regions. Although they cannot completely represent the situations of the 2 regions, the study still has important guiding significance. In the future, if possible, more hospital data should be included in additional in-depth population-based epidemiological studies.

After the pandemic, the number of emergency department visits at UNION did not return to the normal level. On the 1 hand, patients in the region may have remained concerned about SARS-CoV-2 exposure in hospital settings; on the other hand, the health system may not have fully recovered from the crisis. The same situation was not observed at PUTIAN, possibly because the number of patients infected with the virus during the pandemic was small and the medical system did not collapse; thus, after a period of adjustment, the situation quickly returned to normal.

The composition of various diseases in the emergency department prior to the pandemic was basically stable, with some diseases showing slight fluctuations. There was a dramatic increase in the proportion of peptic ulcers after the pandemic, and this was observed in both hospitals. In Wuhan, the first city to report an outbreak, the health care system was severely affected, and for some time, the health care system was in a state of collapse. PUTIAN, although not located in a severely affected area, also experienced a period of social restrictions and community lockdowns during the pandemic. Fear that the health system will collapse or that it will collapse again after the resumption of normal medical practice and the additional virus testing procedures during the treatment process may lead to a great psychological burden on citizens. While the total number of hospitalizations decreased, the number of peptic ulcer cases increased not only in proportion but also in number, so the increase in the relative percentage of hospitalizations for peptic ulcers was unlikely to be due to the lower incidence of hospitalizations for other types of diseases. The results of the increased incidence of peptic ulcers observed in our study may be related to stress. Another explanation may be that patients with relatively mild symptoms did not seek medical attention early in the pandemic, resulting in a larger number of more serious cases later. It has also been demonstrated that^[17,18]^ the number of selective endoscopies decreased sharply during the pandemic period and potentially compromised the early detection of cancers. The main focus of these studies is the direct impact of the pandemic on the medical system by comparison between the period of the pandemic and the same period in previous years. The focus of our study was the social restrictions imposed during the pandemic and the potential impact of the pandemic itself on the medical system after the pandemic. We analyzed gastric malignancies and peptic ulcers separately, and we found a difference. No significant difference was found in the number of malignant tumors before and after the pandemic, so it seems that the number of benign peptic ulcers was affected, which is in line with previous literature reports that peptic ulcer patients are more susceptible to large-scale disasters. From the perspective of biological characteristics, malignancy is affected more by its own proliferative and invasive properties than by stress psychology.

Our previous study^[19]^ showed the effects of viral infections on the digestive system. Medical colleagues from the Kingdom of Saudi Arabia have also reported such effects.^[20]^ However, all the patients in this study were negative for SARS-CoV-2 infection, so peptic ulcers were not directly caused by the virus. Of course, some researchers have already found that in addition to symptoms caused by infection, indirect effects of the pandemic are also prevalent. For example, the breakdown of health systems, social restrictions, and the closure of cities or communities during pandemics indirectly change the behavior of citizens and indirectly affect the incidence of some diseases.^[21]^ French researchers found a dramatic increase in nontraumatic out-of-hospital cardiac arrest cases in Paris during the pandemic.^[14]^ An increase in the incidence of peptic ulcers was also observed after the Japanese earthquake.^[22]^ Therefore, for the public, the stress associated with a large, catastrophic event is high, and it is likely to remain at a high level for some time. Our study showed similar results, with dramatic increases in the incidence of peptic ulcers in different geographic areas in the seven months after control of the pandemic, which may be related to social stress. Therefore, we suggest that the relevant departments should provide psychological counseling to the public in a timely manner. We analyzed the incidence among age groups, but there was no significant difference; it seemed that all adult patients were affected.

To date, the virus is still being transmitted worldwide, but it is believed that virus transmission will gradually be controlled in the near future as many countries begin providing vaccinations. China, supported by a strict lockdown policy and highly effective epidemiological investigations, has basically contained the pandemic. This does not mean, however, that the longer-term effects of the pandemic can be ignored. Over the past year, small outbreaks in some parts of China have required lockdown measures and strict social restrictions to control community transmission. Therefore, health care system personnel still need to pay attention to the lasting effects of the pandemic and adjust policies and balance the allocation of health resources accordingly, including reallocating emergency resources, in hospitals in affected regions. These results could inform the allocation of health resources in the aftermath of the pandemic, including the mobilization of medical supplies for stress-related illnesses and the provision of as many psychologically competent health workers as possible to cope with this new situation. Additionally, it is necessary to provide psychological counseling for the masses, especially those who have been patients.

This study has some limitations. First, both hospitals were major emergency centers in the region, but they were not the only emergency centers, and the study was not a demographic-based observational study, so the results could be biased. Second, the study was limited to 2 regions and is not fully representative of national or global conditions, so it is necessary to integrate comparable data from other regions or countries in the future to further confirm these findings and explore the pathogenesis of the disease and effective prevention measures. Third, all the patients in this study were adults aged more than 18 years, so it is not known whether the same results would be found in adolescents. Fourth, this study was limited to emergency cases involving the digestive system, and the effects on other organ systems are unknown.

## 5. Conclusions

An increase in the incidence of severe peptic ulcer was observed after the pandemic compared to the same period before the pandemic. Therefore, these factors should be considered in the formulation of public health strategies and the allocation of medical resources in the postpandemic era.

## Author contributions

**Conceptualization:** Chenxing Jian, Chunkang Yang, Xiaogang Shu.

**Data curation:** Chenxing Jian.

**Formal analysis:** Chenxing Jian, Zili Zhou.

**Funding acquisition:** Chenxing Jian, Xiaogang Shu.

**Methodology:** Chenxing Jian, Zili Zhou, Chunkang Yang, Ning Zhao, Haijun Bao, Shengbo Han, Jinhuang Chen, Xiaogang Shu.

**Project administration:** Chenxing Jian, Zili Zhou, Jinhuang Chen, Xiaogang Shu.

**Resources:** Chenxing Jian, Jinhuang Chen, Xiaogang Shu.

**Supervision:** Chenxing Jian, Zili Zhou, Chunkang Yang, Jinhuang Chen, Xiaogang Shu.

**Validation:** Chenxing Jian, Xiaogang Shu.

**Visualization:** Chenxing Jian, Zili Zhou, Jinhuang Chen.

**Writing – original draft:** Chenxing Jian.

**Writing – review & editing:** Chenxing Jian, Zili Zhou, Jinhuang Chen.
